# The role of RNA modification in urological cancers: mechanisms and clinical potential

**DOI:** 10.1007/s12672-023-00843-8

**Published:** 2023-12-20

**Authors:** Xuming Zhou, Hezhen Zhu, Cong Luo, Zhaojie Yan, Guansong Zheng, Xiaofeng Zou, Junrong zou, Guoxi Zhang

**Affiliations:** 1 First Clinical College, Gannan Medical University, Ganzhou, 341000 China; 2 Department of Urology, First Affiliated Hospital of Gannan Medical University, Ganzhou, 341000 China; 3 Institute of Urology, First Affiliated Hospital of Gannan Medical University, Ganzhou, 341000 China; 4 Jiangxi Engineering Technology Research Center of Calculi Prevention, Ganzhou, 341000 China

**Keywords:** RNA modification, m^6^A, Prostate cancer, Bladder cancer, renal cancer, Testicular cancer, Biomarkers

## Abstract

RNA modification is a post-transcriptional level of regulation that is widely distributed in all types of RNAs, including mRNA, tRNA, rRNA, miRNA, and lncRNA, where N6-methyladenine (m^6^A) is the most abundant mRNA methylation modification. Significant evidence has depicted that m^6^A modifications are closely related to human diseases, especially cancer, and play pivotal roles in RNA transcription, splicing, stabilization, and translation processes. The most common urological cancers include prostate, bladder, kidney, and testicular cancers, accounting for a certain proportion of human cancers, with an ever-increasing incidence and mortality. The recurrence, systemic metastasis, poor prognosis, and drug resistance of urologic tumors have prompted the identification of new therapeutic targets and mechanisms. Research on m^6^A modifications may provide new solutions to the current puzzles. In this review, we provide a comprehensive overview of the key roles played by RNA modifications, especially m^6^A modifications, in urologic cancers, as well as recent research advances in diagnostics and molecularly targeted therapies.

## Introduction

All urological cancers account for 13% of incidence and 18% of mortality globally. Prostate cancer (PCa) is one of the most common cancers in men and in urology, accounting for 56% of all urological cancers, followed by bladder cancer (BCa), ranked the second most common and fatal cancer, with approximately four times higher mortality in men compared to in women. Renal cell carcinoma (RCC) has an incidence and mortality rate of 17% and 23%, respectively [1–3], while testicular cancer (TC) is the most common malignancy in men aged 15–35 years, with germ cell tumors (GCT) accounting for the majority of TC [4]. Surgery has been regarded as the most effective treatment strategy for urological tumors, but the five-year survival rates remain unsatisfactory [5, 6]. Similarly, androgen deprivation therapy (ADT), combined with a novel endocrine adjuvant therapy, is often employed for locally progressive and metastatic prostate cancer [7–9]. Targeted therapies at the molecular level have changed the management paradigm for patients with RCC, with different regimens developed and approved for treating patients with advanced RCC. However, their realization remains limited because of various factors [10–13]. BCa patients have a higher recurrence rate, are prone to distant metastases, and have a poorer prognosis [14]. Similarly, cisplatin resistance-elicited deaths in TC patients due to metastatic disease are still the main challenge [15]. Although many studies have been conducted to advance our understanding of urological cancers, they are still insufficient to explain the specific mechanisms clearly and provide good treatment strategies.

**Fig. 1:**
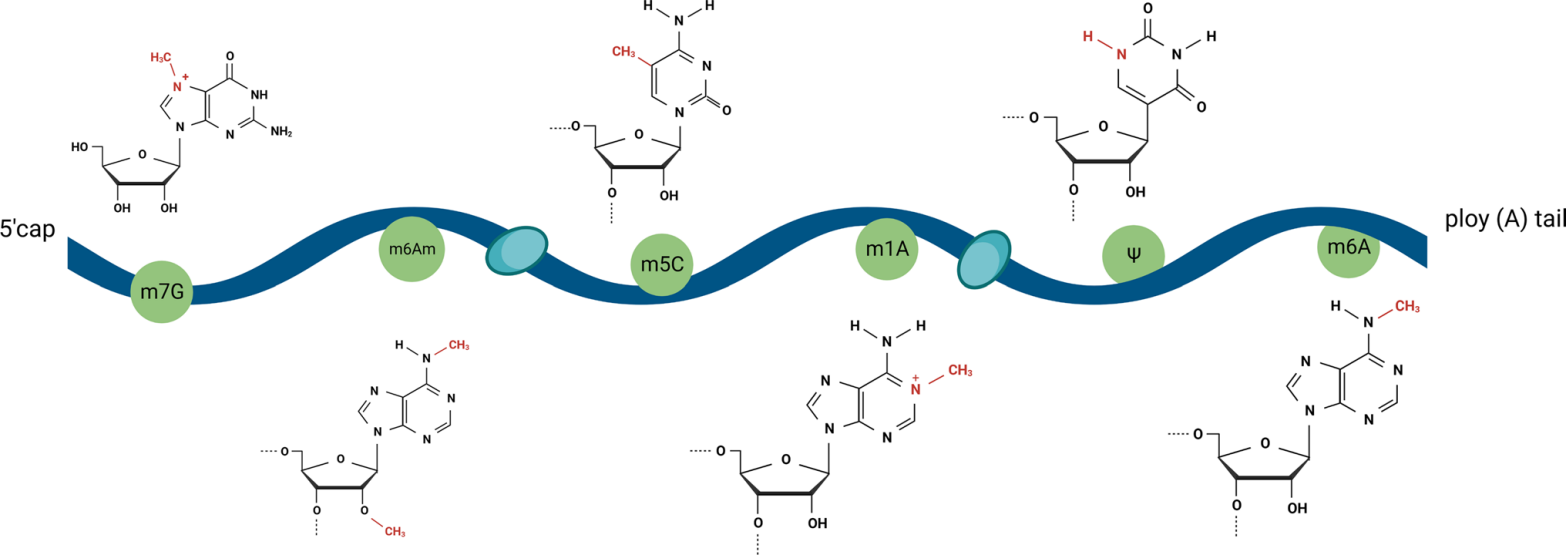
RNA internal methylation modifications. Schematic representation of six internal RNA methylation modifications (m^7^G, m^6^Am, m^5^C, m^1^A, Ψ, and m^6^A) in mRNA. The chemical structures of the methylated nucleotides and the highlighted methyl groups are indicated in the diagram

Transcriptional, post-transcriptional, and post-translational modifications are three levels of epigenetic modifications and changes, including DNA methylation, histone modifications, and chromatin remodeling [16, 17]. Post-transcriptional regulation includes RNA modifications and non-coding RNAs [18], and more than 170 RNA modifications have been identified to date [19], among which m^6^A,5-methylcytosine (m^5^C), N1-methyladenosine (m^1^A), 2'-O methylation (m^6^Am), pseudouridine (Ψ) and N7-Methylguanosine (m^7^G) are the most studied RNA epigenetic modifications [20–25] (see Fig. 1). They have been reported to regulate several critical cellular functions through their regulatory factors during development and disease. Although RNA modifications were discovered as early as the 1990s, RNA modifications still face several understanding gaps [26–29]. With the discovery of RNA demethylases and the application of methylation RNA immunoprecipitation sequencing technology, the role of RNA modifications in physiology and pathology has become a research hotspot [30].

m^6^A is one of the most common internal modifications in messenger RNA (mRNA) and is available in many species [31, 32]. It regulates the self-renewal of embryonic stem cells and cancer cells and facilitates survival after heat shock and DNA damage [31, 33, 34]. The occurrence of m^6^A modifications in the transcriptome was not random. The m^6^A modification sites have the typical recognition sequence DRACH (D = G, A, or U; R = G or A; H = A, C, or U) and are enriched in the coding sequence region (CDS) and 3'UTR [35]. m^6^A is present in almost all types of RNAs, including mRNAs, ribosomal RNAs (rRNAs), long-stranded non-coding RNAs (lncRNAs), miRNAs, small nuclear RNAs (snRNAs), and circular RNAs (circRNAs), and it is dynamically regulated in many physiological and pathological processes, including cancer [36–43].

In this review, we will discuss the role of RNA modifications in urologic cancers. We summarize recent studies that elucidate the therapeutic potential of targeting their aberrant deposition in cancers. m^6^A modification, one of the most widely studied modifications, embodies an important role in the development and progression of urologic cancers. Therefore, we will focus on the key role played by m^6^A in urologic cancers.

## RNA methylation modifications

RNA methylation is a chemical modification in which methyl adenine of RNA is selectively added to methyl groups, catalyzed by methyltransferases [44]. In biology, methylation causes epigenetic changes and regulates their expression but does not affect gene sequences, which can occur in DNA, RNA, and proteins [45]. In this section, we introduce common types of RNA methylation other than m^6^A.

### m^5^C modification

The m^5^C modification was first discovered in the 1970s by adding a living methyl group (mainly S-adenosylmethionine) from a donor to the carbon 5 position of cytosine in RNA [46]. Studies initially focused on tRNA and rRNA for m^5^C modifications [47], wherein the former is involved in optimizing codon and anticodon pairing, maintaining homeostasis, regulating stress responses, and controlling translation efficiency and accuracy [48–53], and plays a vital role in enhancing bacterial drug resistance [54, 55]. Similar to m^6^A methylation, regulatory RNA m^5^C levels can be classified by function into "writers,” "erasers," and "readers " proteins.

The m^5^C methylation "writers" protein of human RNA mainly comprises the NSUN family and DNMT2 [56]. The NSUN family catalyzes methyl transfer via a shared mechanism of covalent binding between the cysteine of the methyltransferase and cytosine in RNA to form a covalent intermediate, followed by nucleophilic addition of the electron-rich cytosine ring to the methyl group on S-adenosylmethionine (SAM) to complete methylation [57]. The most extensively studied RNA m^5^C methyltransferases in recent years are the NSUN family proteins, comprising nine members. Several have catalytic and release sites for methylation transferases, the most prominent and well-defined of which is NSUN2 [21]. SSUN2 is encoded by the NSUN2 gene on chromosome 10 and is a nucleolar RNA methyltransferase that catalyzes the m^5^C methylation of tRNA, mRNA, and ncRNA [58]. The SSUN2 mainly depends on two cysteine sites to function as a methyltransferase, with the C321 site catalyzing the methylation of cytosine by binding to the pyrimidine ring of cytosine to form a covalent bond and the C271 site mediating the release of RNA after methylation. The intracellular localization of NSUN2 in human epidermal cells was found to vary in different cell cycles [59, 60]. It is mainly distributed in the nucleus in G1 phase, between the nucleolus and nucleoplasm in S phase, localized in the cytoplasm in G2 phase, and in the centriole in M phase [61].

Similarly, NSUN1 and NSUN3-7 have also been demonstrated to bind RNAs, each catalyzing the methylation of different RNAs. NSUN1 is a nucleoprotein that primarily catalyzes the methylation of yeast 25S rRNA, 60S ribosomal subunit, and 26S rRNA[62]. NSUN3 is localized in the mitochondrial matrix of human and mouse cells and recognizes the anticodon loop of mitochondrial methionine transfer RNA (tRNAMet), methylating the C34 site [63]. NSUN4 is an rRNA-specific RNA methyltransferase that mainly acts on 12S rRNA in eukaryotic mitochondria [64, 65], while NSUN5 is a methyltransferase responsible for modifying the second m^5^C position in eukaryotic rRNAs and maintaining its high-level structure by catalyzing 28S and 25S rRNA methylation in nematodes, Drosophila, yeast, and plants [66]. NSUN6 is present in the eukaryotic cytoplasm and is partially localized in the Golgi apparatus, catalyzing the methylation of the 3'-UTRs C72 site of tRNA(Cys) (cysteinyl transfer RNA) and tRNA(Thr) (threonine transfer RNA) [67]. In contrast, NSUN7 silencing in human hepatocytes significantly reduced cytosine methylation levels of PFKL, SIRT5, IDH3B, and HMOX, suggesting that NSUN7 may act on eukaryotic eRNAs [68]. DNMT2 is also an m^5^C methyltransferase that improves tRNA stability and influences the expression and precision of protein synthesis, thereby ensuring precise peptide synthesis by recognizing near-homologous codons [50, 53, 69].

The process of DNA demethylation and TET family of demethylases are well known. The primary recognized mechanism is that TET catalyzes the m^5^C demethylation on DNA under the synergistic action of α-ketoglutarate and divalent iron ions [70–72]. It was recently found that knocking down TET2 in mouse embryonic stem cells resulted in a significant decrease in hm^5^C levels on tRNA, while its overexpression resulted in increased hm^5^C levels and decreased m^5^C levels on tRNA, and in vitro studies have revealed that oxidation of m^5^C modifications on tRNA catalyzed by TET2 also promoted the translation process [73]. The hydroxylation of m^5^C modification at the C34 site of mitochondrial tRNA to hm^5^C was also found to be mediated by another demethylase, ALKBH1, where its knockdown can result in impaired mitochondrial translation and respiratory function [74].

The reported m^5^C methylation recognition proteins mainly include ALYREF, RAD52, and YBX1, where ALYREF regulates nucleation, RAD52 is involved in DNA damage repair, and RNA stability is regulated by YBX1 [75]. ALYREF, a recognition protein for m^5^C methylation, can specifically bind to mRNAs with m^5^C modifications in the nucleus to form a complex of mRNPs to promote mRNA outgrowth. In ALYREF knockdown cell lines, mRNAs with mC modification accumulated in the nucleus, which ALYREF could only abolish in the back-complemented wild type. In contrast, ALYREF in the back-complemented mutant type remained unchanged, further verifying that ALYREF promotes mRNA outgrowth by recognizing and binding to mRNAs with mC modifications [75]. YBX1 is a well-known multifunctional DNA- and RNA-binding protein found in early zebrafish embryos that recognizes and binds m^5^C-modified mRNA through the Trp45 residue of the YBX1 cold excitation domain [76, 77].

### m^1^A modification

N1-methyladenine (m^1^A) is an essential post-transcriptional RNA modification formed by adding methyl to the N1 position of adenosine [22]. The m^1^A methylation modification was discovered in the 1960s [78], which was previously thought to occur mainly in rRNA and tRNA, in maintaining RNA tertiary structure and affecting protein translation efficiency [79–81]. Recently, high-throughput sequencing has revealed that m^1^A modifications are also present in mRNA and can interfere with the Watson–Crick base-pairing principle, suggesting an important regulatory role for m^1^A modifications [82, 83].

The m^1^A-modified "writer" proteins included TRMT6, TRMT61A, TRMT61B, TRMT10C, and NML. TRMT61A, a catalytic subunit, contains a SAM-binding domain forming a functional complex with TRMT6 that binds to tRNA [84, 85], whereas TRMT10C and TRMT61B catalyze m^1^A modifications in mitochondrial tRNA [86, 87]. Recent studies have shown that these tRNA methyltransferases may also catalyze m^1^A mRNA [83, 88]. NML is located in the nucleus and methylesterase on m^1^A of 28S rRNA [89]. ALKBH1, ALKBH3, and FTO can demethylate m^1^A [74, 90, 91]. IYT521-B homolog (YTH) family proteins were recently identified as "readers" of m^1^A modifications. However, they also play a major role in m^6^A modifications [88, 92]. These include YTHDF1/2/3 and YTHDC1, which bind to the m^1^A site with weaker affinity than m^6^A; hence, their function as m^1^A readers still require further investigation [22].

### 2'-O methylation (m^6^Am)

The m^6^Am methylation modification is a chemical modification in which rRNA is methylated at the 2' position by RNA methylesterase [23]. m^6^Am methylation modifications are widely distributed in mRNA, tRNA, rRNA, miRNA, and other molecules [93]. It has been disclosed that m^6^Am methylation affects the binding of mRNA to proteins, regulates the translation efficiency of rRNA, and participates in biological processes, such as tRNA recognition [94]. Recent studies have identified phosphorylated CTD interaction factor 1 (PCIF1) as a methyltransferase of m^6^Am [95–97], in addition to FTO, which also acts as a demethylase of m^6^Am [91, 98].

### m^7^G modification

m^7^G is one of the most abundant modifications in tRNA, rRNA, and mRNA, playing important roles in regulating RNA processing, metabolism, and function [25] and also occurs in miRNAs [99, 100]. In mammals, the METTL1 regulates m^7^G, which binds to the corresponding WD repeat domain 4 (WDR4), mediating m^7^G modifications in tRNA, miRNA, and mRNA [101]. RNA guanine-7 methyltransferase (RNMT) and its cofactor RNA guanine-7 methyltransferase (RAM) are involved in m^7^G modification at the 5' cap end of mRNA [102]. It has been reported that Williams-Beuren syndrome chromosome region 22 (WBSCR22) and tRNA methyltransferase activator subunit 11–2 (TRMT112) mediate m^7^G methylation in rRNA [103].

### Ψ, Pseudouridine

Pseudouridine is the "fifth nucleotide" of RNA and is the most abundant RNA modification generally produced by uridine isomerization [104–106]. The pseudouridine of mRNA performs codon alteration, splicing, transcript stability enhancement, peptide bond formation, and stress responses [104, 107–110]. The pseudouridylation of RNA in eukaryotes mainly follows two pathways, that is, an RNA-dependent mechanism catalyzed mainly by DKC1, which forms a complex with box H/ACA snRNA, pseudouridylates RNA, and mediates post-transcriptional modifications of RNA, and an RNA non-dependent mechanism, which is directly recognized and catalyzed by an independent pseudouridine synthase substrate [111–114]. Specific "eraser" and "reader" for Ψ have still not been discovered [115, 116].

## m^6^A modification

**Fig. 2:**
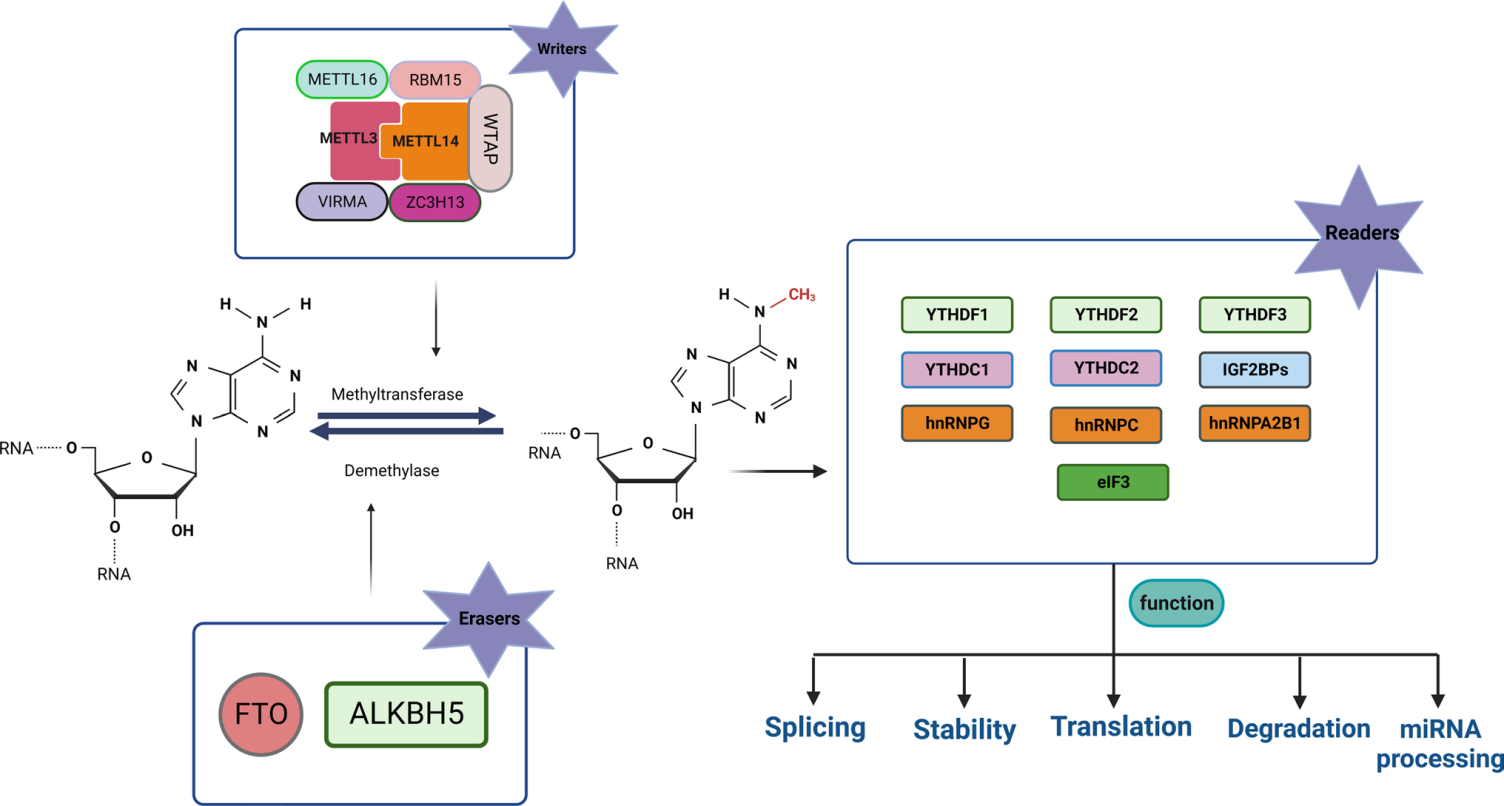
m^6^A RNA methylation and m^6^A modification mechanism. METTL3, METTL14, and WTAP form the core component of the methyltransferase complex and catalyze the methylation of N6 adenosine with other regulatory cofactors VIRMA, RBM15, ZC3H13, and METTL16. However, the deposition of m^6^A is reversible and dependent on the demethylases FTO and ALKBH5. m^6^A binding proteins can also recognize m^6^A. YTHDC1 can alternative splicing and RNA export; YTHDF1/2/3, eIF3 regulates RNA translation and degradation. IGFBP1/2/3 promotes RNA stability. hnRNPG/C and hnRNPA2B1 can regulate mRNA splicing.

The m^6^A, a well-known post-transcriptional modification, was first discovered in 1974 and is the most abundant internal modification in mRNA, with approximately 25% of mRNAs carrying at least one m^6^A site [26, 117, 118]. m^6^A modifications are usually enriched at 3'UTRs, near-stop codons, long inner exons, intergenic regions, introns, and 5′ UTRs [119, 120]. Most approaches to m^6^A detection rely on the immunoprecipitation of methylated RNA using m^6^A recognition-specific antibodies, followed by a UV cross-linking step to bind the methylated RNA to the antibody, thereby allowing recognition of the m^6^A site [35, 119, 121, 122]. The mechanism of m^6^A methylation modification regulation is a dynamic process, catalyzed by m^6^A methyl transfer "writers,” removed by "erasers,” and finally recognized and bound by m^6^A "readers" to direct the translation and degradation of downstream mRNAs (See Fig. 2 and Table 1).

**Table 1:** m^6^A modification-related factors

Type	Factors	Function	Refs.
Writer	METTL3/14, WTAP, VIRMA, RBM15	It catalyzes the m^6^A modification of adenylate on mRNA	[128, 129, 131, 132]
	METTL16	Methylated snRNA, pre-mRNA, and ncRNA	[130]
	ZC3H13	Connecting WTAP and RBM15	[133]
Eraser	FTO	Demethylation of m^6^A	[135]
	ALKBH5	Demethylation of m^6^A	[136]
Reader	YTHDC1	Alternative splicing and RNA export	[143]
	YTHDC2	mRNA degradation and translation initiation	[144]
	YTHDF1	Promote translation	[141]
	YTHDF2	Promotes RNA degradation	[142]
	YTHDF3	Promotes mRNAs translation and degradation	[141]
	IGFBP1/2/3	Promotes RNA stability	[143]
	HnRNPG/C	Regulation of mRNA splicing	[41, 145]

### m^6^A “writers”

m^6^A methylation is catalyzed by the methyltransferase complex (MTC), which catalyzes the methyl transfer of S-adenosylmethionine (SAM) to the nitrogen atom at position 6 of adenine [123]. The core component of MTC is methyltransferase-like protein 3 (METTL3), and the remaining components include METTL14, METTL16, WTAP, VIRMA, RBM15, and ZC3H13 [124–127]. METTL3 possesses a methyltransferase structural domain (MTD) that binds and catalyzes the transfer of methyl in SAM to the adenine base of RNA for the protein production of methionine homocysteine (SAH) [128]. Both METTL14 and METTL3 synergistically induce m^6^A modification [129]. The methyltransferase structural domain of METTL16 contains the Rossmann-like fold of class I methyltransferases with SAM as the methyl donor [130], whereas WTAP serves as an important cofactor for MTC [131]. VIRMA is involved in the recruitment and direction of core methyltransferase components to specific regions of mRNA [132], and ZC3H13 assists WTAP-RBM15 complex localization to the nucleus [133].

### m^6^A “erasers”

m^6^A demethylation removes m^6^A methylation modifications and co-regulates m^6^A modifications with m^6^A methyltransferases to achieve a dynamic balance in m^6^A methylation modification levels in vivo [124, 134]. The two identified demethylases include FTO and ALKBH5, two mutually independent demethylases localized in the nucleus and belonging to the α-ketoglutarate dioxygenase family, catalyzing m^6^A demethylation in a Fe(II)- and α-ketoglutarate-dependent manner, respectively. Mechanistically, m^6^A is oxidized to N6-hydroxymethyl nonanoic acid (hm^6^A), which is then converted to N6-formyl adenosine (f6A), which is finally converted to adenosine [135]. FTO can catalyze the demethylation of m^6^A modification in addition to m^6^Am, reducing m^6^A and m^6^Am methylation levels on mRNA [136].

### m^6^A “readers”

The m^6^A methyl recognition protein selectively recognizes and binds to m^6^A methylation modifications in target RNAs and participates in various stages of downstream target RNA metabolism [137–139]. The YTH family proteins include m^6^A YTH binding protein 1/2/3 (YTHDF1/2/3) and YTH structural domain protein 1/2 (YTHDC1/2) [140], where YTHDF1 and YTHDF3 interact through the translation of eIF3 and eIF4A3, resulting in increased translation efficiency of mRNAs [141]. YTHDF2 relocates, translating mRNAs from the cytoplasm to intracellular mRNA degradation sites and promoting mRNA degradation [142]. Similarly, YTHDC1 binds to sites on hnRNA where m^6^A methylation modifications occur, facilitating hnRNA splicing and processing of mRNA and facilitating mRNA export from the nucleus [143]. YTHDC2 selectively binds to m^6^A modification sites in the typical sequence of m^6^A and enhances the translation efficiency of target mRNAs [144]. hnRNP superfamily proteins include hnRNPA2B1 and hnRNPC/G [145], where the former recognizes m^6^A modifications in primary miRNA transcripts and interacts with DGCR8 to facilitate the processing of miRNAs [36]; the latter does not directly bind to m^6^A modification sites, but recognizes methylation modifications and then regulates mRNA abundance and splicing [41, 145]. By recognizing m^6^A modifications under normal and stress conditions, IGF2BP1/2/3 can increase mRNA stability and translational capacity [146].

## Role of m^5^C, m^1^A, m^6^Am, m^7^G, and Ψ modification in urological cancers

Recently, there has been a gradual increase in research on m^5^C and cancer, although the specific mechanism of action of m^5^C in some cancers remains unclear. In urological cancers, m^5^C is also in its infancy, and current and future studies will continue to explore the role and molecular mechanisms of m^5^C. Currently, models of m^5^C-related regulators for predicting cancer prognosis, including PCa, RCC, and BCa, are constantly being established [147–150]. It has been reported that the m^5^C methyltransferase NSUN2 is highly expressed in prostate cancer tissues and is associated with poor patient prognosis. Mechanistically, NSUN2 stabilizes the AR post-transcriptionally through m^5^C-YBX1-dependent m^5^C modifications. AR acts as a transcription factor that regulates NSUN2 transcription [151]. In bladder cancer, m^5^C may play a key role in the hypoxia-glycolytic network. The m^5^C RNA-binding protein ALYREF stabilizes PKM2 mRNA in an m^5^C-dependent manner and promotes bladder cancer cell proliferation through PKM2-mediated glycolysis [152].

ALKBH3, a demethylase of m^1^A, is also a potential diagnostic marker for prostate cancer.ALKBH3 is highly expressed in prostate cancer and correlates with disease progression and prognosis [153, 154]. The study of m^1^A in urological cancers is still limited to computer models and needs to be explored in more in vivo and in vitro experiments [155]. Functional RNAi screens in human bladder cancer cells and mouse models have identified m^6^Am methyltransferase PCIF1 as a novel tumor suppressor, the first indication of its role for m^6^Am in cancer [156]. Studies have shown that m^7^G modification is significantly involved in the development and progression of urological cancers. The m^7^G methyltransferase METTL1 is highly upregulated in BCa tissues, and its expression is positively correlated with clinically advanced, high-grade tumors. METTL1 plays an oncogenic role in BCa development and progression. Functional experiments have shown that METTL1 deletion effectively inhibits BCa proliferation, migration, and invasion, both in vivo and in vitro. Mechanistically, METTL1 mediates specific RNA translation by altering the m^7^G modification of tRNA and inhibiting ribosomal pausing during tRNA-mRNA codon recognition [157]. In addition, METTL1 expression is upregulated in RCC and PCa [158]. m^7^G studies in urological cancers still need to be validated by more functional experiments. Studies on pseudouridine in urological cancers have revealed the predictive value of Ψ in these cancers. For example, the methyltransferase DKC1 of Ψ is elevated in PCa and is expected to be a novel biomarker for PCa [107, 159].

## Role of m^6^A modification in urological cancers

**Fig. 3:**
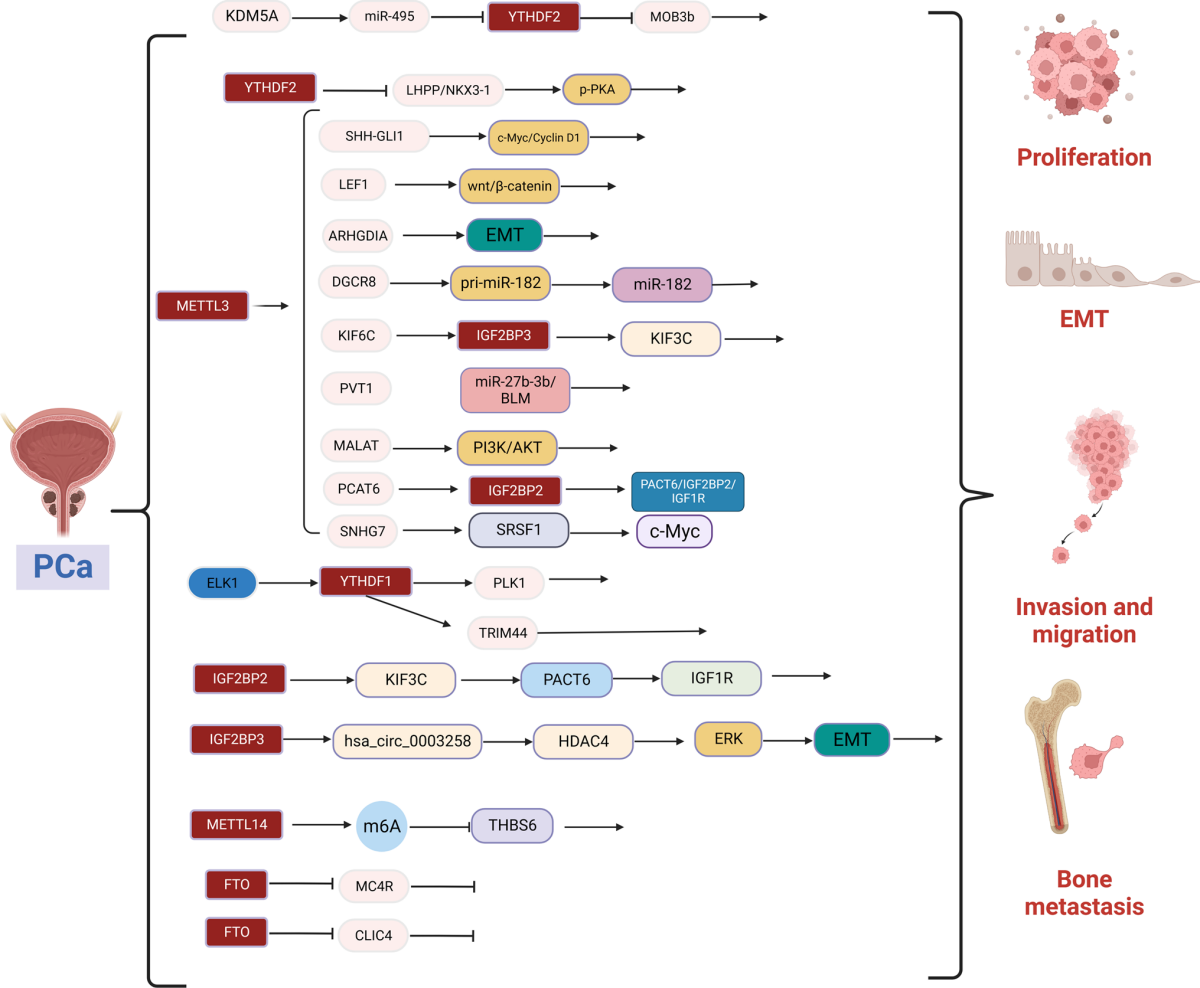
Molecular mechanisms by which m^6^A modifications regulate the biological functions of PCa. Writer proteins METTL3 and METTL14 are involved in PCa proliferation, invasion, migration, bone metastasis, angiogenesis, and glycolysis. Eraser protein FTO inhibits PCa proliferation, invasion, and migration. Reader proteins YTHDF1/2 and IGF2BP2/3 are involved in PCa proliferation, invasion, migration, and bone metastasis

**Fig. 4:**
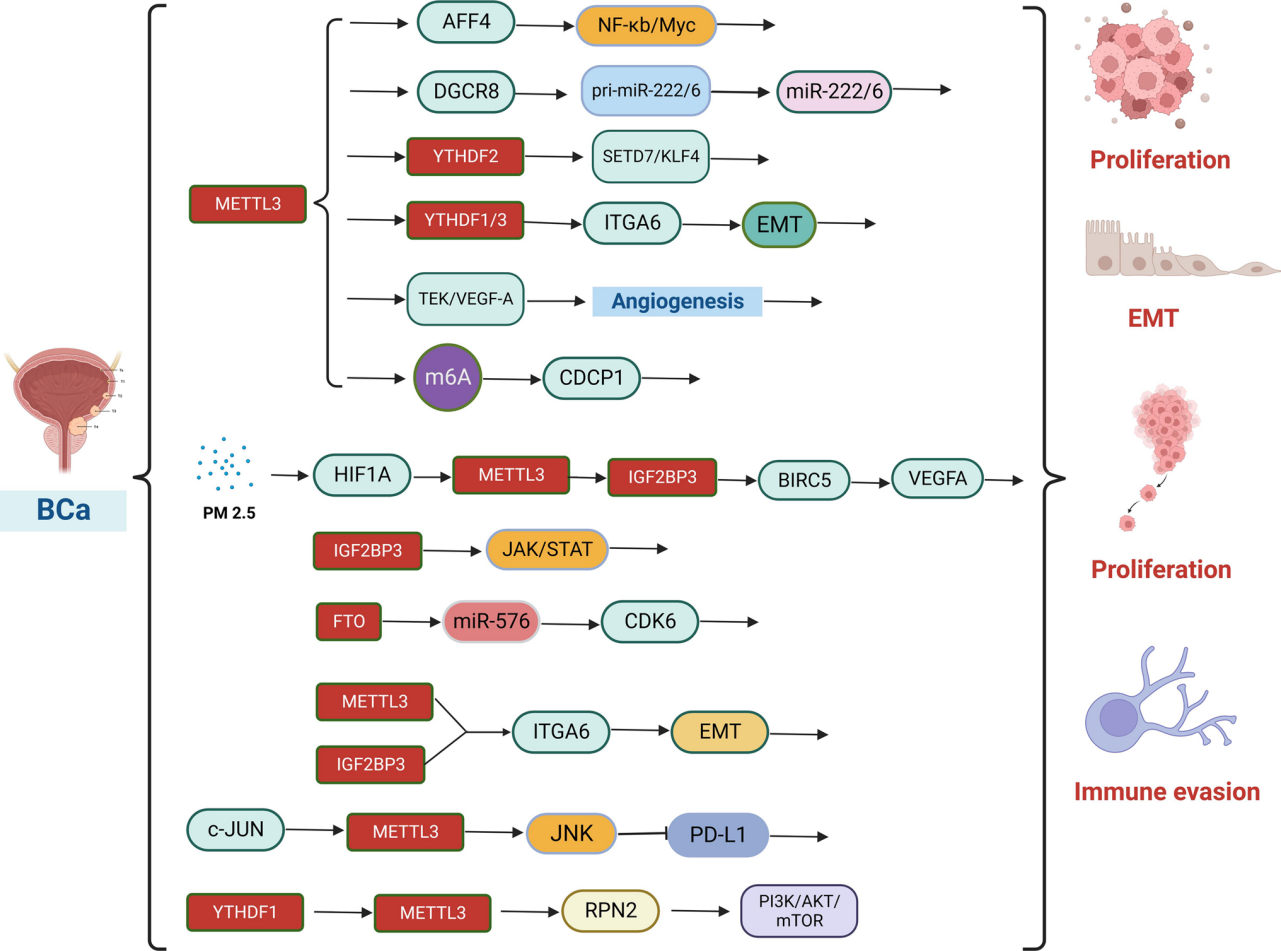
Molecular mechanisms by which m^6^A modifications regulate the biological functions of BCa. Writer proteins METTL3 and METTL14 are involved in BCa proliferation, invasion, migration, angiogenesis, cell adhesion and immune escape. Eraser protein FTO is involved in BCa proliferation, invasion, and migration. Reader proteins YTHDF1 and IGF2BP3 are involved in BCa proliferation

**Fig. 5:**
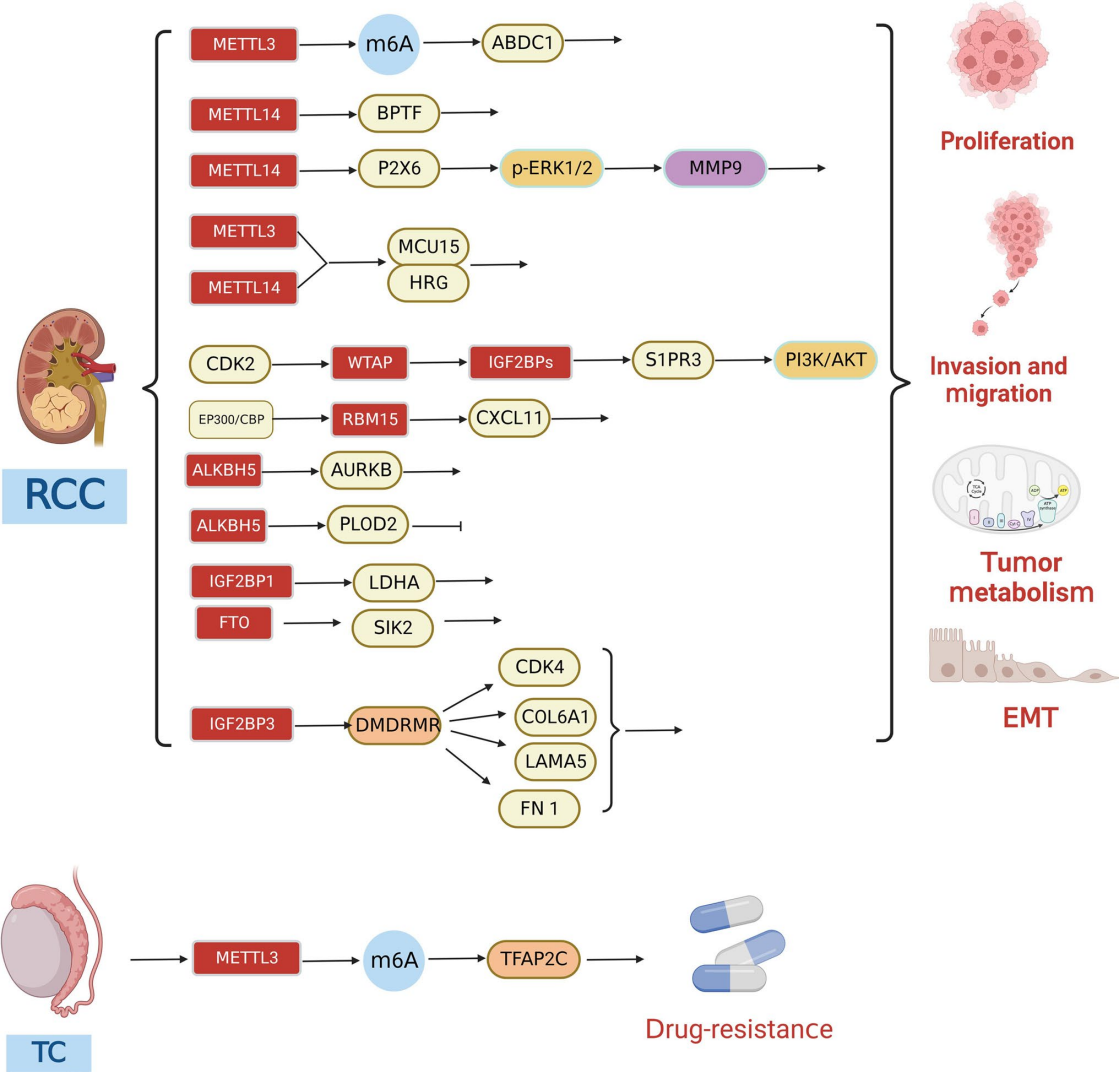
Molecular mechanisms by which m^6^A modifications regulate the biological functions of RCC and TC. Writer proteins METTL3, METTL14, WTAP, and RBM15 are involved in RCC proliferation, invasion, and migration. Eraser proteins FTO and ALKBH5 are involved in RCC proliferation, invasion, migration, and autophagy. Reader proteins IGF2BP1/3 are involved in RCC proliferation and energy metabolism. METTL3 is involved in TC drug resistance

m^6^A methylation modifications and their molecular functions have been studied in many human diseases, particularly the role of m^6^A-mediated regulation of gene expression in cancer. Different signaling pathways mediate the translational machinery to fulfill anabolic demands in cancerous tissues. Many studies have also depicted that m^6^A can play a role in the biology of urological cancers via multiple molecular pathways (see Figs. 3,4,5 and Table 2).

**Table 2:** RNA modifying regulators of m^6^A in Urologic cancers

Cancer type	Category	m^6^A regulations	expression	property	Biological functions	Mechanism	Signaling pathways	Related genes	Refs.
PCa	Writer	METTL3	Up	Oncogene	Proliferation, invasion, and migration	Inhibition expression of LHPP mRNA and NEX3-1 mRNA	AKT	YTHDF2, LHPP, NEX3-1	[167]
	Writer	METTL3	Up	Oncogene	Proliferation	Enhance expression of GLI1 mRNA	c-Myc/Cyclin D1	SHH, GLI1	[160]
	Writer	METTL3	Up	Oncogene	Proliferation	Enhance expression of LEF1 mRNA	Wnt/β-catenin	LEF1	[168]
	Writer	METTL3	Up	Oncogene	Proliferation, invasion, and migration	Enhance expression of ARHGDIA mRNA	EMT	ARHGDIA	[169]
	Writer	METTL3	Up	Oncogene	Proliferation	Promote the maturation of pri-miR-182	Wnt/β-catenin	DGCR8, miR-182	[170]
	Writer	METTL3	Up	Oncogene	Invasion and migration	Enhance KIF6C mRNA stability	IGF2BP3/KIF3C	KIF3C, KIF6C, IGF2BP3	[171]
	Writer	METTL3	Up	Oncogene	Proliferation	Enhance expression of MALAT mRNA	PI3K/AKT	MALAT	[172]
	Writer Writer Writer	METTL3 METTL3 METTL3	Up Up Up	Oncogene Oncogene Oncogene	Proliferation Bone metastasis Glycolysis	Enhance expression of PVT1 mRNA Enhance expression of PCAT6 mRNA Enhance SNHG7 mRNA stability	miR-27b-3b/BLM PCAT6/IGF2BP2/IGF1R c-Myc	PVT1 IGF2BP2, IGF1R SNHG7, SRSF1	[173] [174] [178]
	Writer	METTL14	Up	Oncogene	Proliferation, invasion, and angiogenesis	Inhibition expression of THBS6 mRNA	/	THBS6	[180]
	Eraser Eraser	FTO FTO	Down Down	Suppression Suppression	Proliferation Proliferation, invasion, and migration	Inhibition expression of MC4R Reduced degradation of CLIC4 mRNA	/ /	MC4R CLIC4	[183] [184]
	Reader	YTHDF1	Up	Oncogene	Proliferation, invasion, and migration	Enhance expression of PLK1	PI3K/AKT	ELK1, PLK1	[188]
	Reader	YTHDF1	Up	Oncogene	Proliferation, invasion, and migration	Enhance expression of TRIM44	/	TRIM44	[189]
	Reader	YTHDF2	UP	Oncogene	Proliferation, invasion, and migration	Induction of MOB3b mRNA degradation	KDM5a/miRNA-495/YTHDF2/ m^6^A-MOB3b	KDM5a, miR-495, MOB3b	[190]
	Reader	IGF2BP2	Up	Oncogene	Proliferation, bone metastasis	Enhance expression of PCAT6 mRNA, Enhance IGF1R stability	PCAT6/IGF2BP2/IGF1R	PCAT6, IGF1R	[174]
	Reader	IGF2BP3	Up	Oncogene	Invasion and migration	Binding of circular RNA hsa_circ_0003258 enhances the stability of HDAC4 mRNA, activates the ERK pathway, and triggers EMT	ERK, EMT	HDAC4, hsa_circ_0003258	[194]
BCa	Writer	METTL3	Up	Oncogene	Invasion and migration	Enhance expression of AFF4 mRNA	NF-κb, Myc	AFF4	[196]
	Writer	METTL3	Up	Oncogene	Proliferation and immune escape	c-JUN binds to the METTL3 promoter, increases the expression of METTL3 and global m^6^A levels, and suppresses PD-L1 expression abundance	JUN	c-JUN	[197]
	Writer/ Reader	METTL3/YTHDF2	Up	Oncogene	Invasion and migration	Degradation of mRNA of tumor suppressors SETD7 and KLF4	METTL3/YTHDF2 m^6^A	SETD7, KLF4	[198]
	Writer	METTL3	Up	Oncogene	Cell adhesion		YTHDF1/3/ITGA6	ITGA6	[199]
	Writer	METTL3	Up	Oncogene	Proliferation and angiogenesis	Enhance expression of TEK and VEGF-A mRNA	/	TEK, VEGF-A	[200]
	Writer	METTL3	Up	Oncogene	Promotes malignant transformation of urinary tract epithelial cells	mediates effective site-specific m^6^A mounting on CDCP1 mRNA	/	CDCP1	[201]
	Writer	METTL3	Up	Oncogene	Proliferation, invasion, migration, and angiogenesis	PM2.5 Induced METTL3 promoter hypermethylation and increased binding affinity of transcription factor HIF1A to enhance METTL3 expression	HIF1A/METTL3/IGF2BP3/BIRC5/VEGFA	HIF1A, BIRC5,VEGFA	[203]
	Writer	METTL14	Up	Oncogene	Proliferation	Enhancing the stability of Notch1 mRNA	Notch	Notch1	[204]
	Eraser	FTO	Up	Oncogene	Proliferation and invasion	Regulation of CDK6 expression	FTO/miR-576/CDK6	MiR-576, CDK6	[206]
	Eraser	FTO	Up	Oncogene	Proliferation and invasion	Regulation of demethylase activity on PYCR1	/	USP18, PYCR1	[207]
	Eraser Reader	FTO YTHDF1	Up Up	Oncogene Oncogene	Proliferation, invasion, and migration Proliferation	Regulation of MALAT1 expression /	MALAT/miR-384/MAL2 YTHDF1-RPN2-PI3K/AKT/mTOR	MALAT1, miR-384, MAL2 METTL3, PRN2	[208] [210]
	Reader	IGF2BP3	Up	Oncogene	Cell cycle and proliferation	/	JAK/STAT	/	[212]
RCC	Writer	METTL3	Up	Oncogene	Tumor migration and stem cell-like tumorigenic cells	Enhance expression of ABCD1 mRNA	/	ABCD1	[214]
	Writer	METTL14	Down	Suppression	Proliferation, invasion, and migration	Regulation expression of BPTF mRNA	BPTF/ENO2/SRC	BPTF, ENO2, SRC	[215]
	Writer	METTL14	Down	Suppression	Proliferation, invasion, and migration	Regulation expression of P2X6 mRNA	P2X6-Ca^2+^-p-ERK1/ERK2-MMP9	P2X6, MMP9	[216]
	Writer	METTL3/14	Down	Suppression	Invasion and migration	Enhance expression of MUC15 and HRG mRNA	/	MCU15, HRG	[217]
	Writer	WTAP	Up	Oncogene	Proliferation, invasion, and migration	Enhancement of S1PR3 mRNA stability and performance in an IGF2BPs-dependent manner	PI3K/AKT	SIRP3, miR-501-3p, CDK2	[217–219]
	Writer Eraser	RBM15 FTO	Up Up	Oncogene Oncogene	Proliferation Inhibits autophagy and promotes proliferation	Enhance expression and stability of CXCL11 Regulation expression of SIK2 mRNA	EP300/CBP/RBM15/CXCL11 /	EP300, CBP, CXCL11 SIK2, IGF2BP2	[221] [224]
	Eraser	ALKBH5	Up	Oncogene	Proliferation, invasion, and migration	Regulation expression of AURKB mRNA	/	AURKB	[222]
	Eraser	ALKBH5	Down	Suppression	Proliferation, invasion, and migration	Regulation expression of PLOD2 mRNA	/	PLOD2	[223]
	Reader	IGFBP1	Down	Suppression	Energy metabolism	Regulation expression of LDHA mRNA	/	LDHA	[231]
	Reader	IGFBP3	Down	Suppression	Proliferation	Enhance activity of CDK4, COL6A1, LAMA5 and FN1	/	CDK4, COL6A1, LAMA5, FN1	[232]
TC	Writer	METTL3	Up	Oncogene	Drug-resistance	Enhance expression and stability of TFAP2C mRNA	/	TFAP2C	[236]

### Role of m^6^A modification in PCa

#### The role of m^6^A writers in PCa

METTL3 was the first identified m^6^A writer and the only catalytic subunit, and its upregulation in PCa translates into playing important roles in cancer progression. METTL3 expression was upregulated in PCa cell lines, where it knockdown-induced apoptosis in cancer cells [160]. METTL3 upregulation was also associated with poor prognosis in PCa patients, as its expression was upregulated in PCa tissues, especially bone metastases [161, 162]. METTL3 was frequently upregulated in PCa as an upstream cooperating factor of YTHDF2. Further analysis of MeRIP-seq, mRNA-seq, and databases identified LHPP and NKX3-1 as the main targets of YTHDF2, while LHPP and NKX3-1 were found to be tumor suppressors regulating tumor progression by inhibiting AKT phosphorylation [163–167], which was also reported by Cai et al. where METTL3 had elevated levels in PCa cells, promoting its growth by regulating the hedgehog pathway [160]. Similarly, METTL3 can also affect Wnt/β-catenin in the Wnt pathway via m^6^A methylation of LEF1 mRNA to promote PCa proliferation and migration [168]. METTL3 is involved in PCa metastasis by mediating epithelial-mesenchymal transition by regulating the expression of ARHGDIA migration-associated protein [169]. Among non-coding RNAs, the METTL3 is imperative for DGCR8 to regulate pri-miRNAs in PCa, where experiments have revealed that m^6^A modification-dependent METTL3 can interact with DGCR8 to enhance the recognition of prior-miR-182 in PCa, thereby promoting the maturation of pri-miRNAs, leading to PCa proliferation, in addition, to mediate m^6^A modification of KIF3C mRNA, thereby promoting PCa progression [170, 171]. METTL3-mediated m^6^A-modified lncRNA MALAT1 can lead to PCa proliferation by activating the PI3K/AKT signaling pathway, and METTL3-mediated lncRNA PVT1 was found to regulate the miR-27b-3b/BLM signaling axis [172, 173]. Lang et al. identified a novel molecular mechanism of bone metastasis in which METTL3-mediated m^6^A modification promotes the upregulation of PCAT6 in an IGFBP2-dependent manner. PACT6 enhances IGF1R mRNA stability through the PACT6-IGF2BP2-IGF1R RNA–protein trimer, thereby upregulating IGF1R expression and promoting bone metastasis and tumor growth in PCa [174]. Glycolysis is the preferred pathway for energy acquisition by cancer cells; however, glycolysis is not a hallmark of primary prostate cancer and plays a critical role only in advanced tumors [175–177]. METTL3 enhances SNHG7 stability by regulating m^6^A modifications of SNHG7 and recruits SRSF1 to regulate c-Myc expression, further promoting glycolysis in PCa cells [178].

Moreover, among other components of methyltransferases, high expression of VIRMA may also be associated with poor prognosis of PCa [179]. It was also discovered that METTL14 promoted PCa proliferation in an m^6^A-dependent manner by inhibiting the expression of THBS6, an angiogenesis-inhibiting glycoprotein [180].

#### Role of m^6^A erasers in PCa

RNA modifications were demonstrated to be reversible with the discovery of FTO and ALKBH5. FTO is commonly downregulated in PCa tissues and cell lines, and patients with lower FTO expression have more advanced tumor stages and higher Gleason scores [181, 182]. Li et al. reported that FTO inhibits PCa progression by downregulating melanocortin receptor 4 (MC4R) expression [183]. Furthermore, downregulation of FTO was associated with a poor prognosis of PCa, and functional experiments demonstrated that FTO depletion promoted PCa proliferation and metastasis in vivo and in vitro. Chloride intracellular channel 4(CLIC4) is a functional target of FTO-mediated m^6^A modifications. FTO inhibits PCa proliferation and metastasis by reducing CLIC4 mRNA in an m^6^A-dependent manner [184]. Similarly, a recent study discovered that ALKBH5 expression is downregulated in PCa tissues, inhibiting the growth of PCa cell lines [185]. Overall, studies on m^6^A erasers in PCa are limited and require further exploration.

#### Role of m^6^A readers in PCa

m^6^A readers also play an important role in PCa progression. YTHDC1 was found to bind and colocalize with oncogene MET adhesin in subnuclear patches and affect PCa proliferation [186]. YTHDC2 expression was upregulated in PCa tissues and cell lines and significantly correlated with PSA levels and Gleason scores, whereas YTHDC2 overexpression promoted proliferation and invasion in PCa cell lines [187]. It was also discovered that YTHDF1/2 was overexpressed in PCa, and PLK1, a critical cell cycle factor, is a direct target of YTHDF1 in PCa cells. Furthermore, ELK1-activated YTHDF1 controls PLK1 translation efficiency in an m^6^A-dependent manner, enabling activation of the PI3K/AKT signaling pathway, leading to PCa progression. YTHDF1 can also contribute to PCa progression by regulating TRIM44 to promote PCa cell proliferation and migration [188, 189]. YTHDF2 enables PCa progression by mediating the degradation of the tumor suppressors LHPP and NKX3-1 and activating the AKT signaling pathway [167]. YTHDF2 also served as a direct target of miR-495 and miR-493-3p. On the lysine demethylase 5a (KDM5a)/miRNA495/YTHDF2/ m^6^AMOB3b axis, YTHDF2 recognizes m^6^A of MOB3b mRNA, inducing MOB3b mRNA degradation and suppresses its expression. The miR-493-3p suppresses YTHDF2 expression, thereby increasing the level of m^6^A [190, 191]. Therefore, high expression levels of YTHDF2 promote the proliferation, migration, and invasion of PCa cells.

hnRNPA2B1 is highly expressed in CRPC cells, promoting proliferation and leading to a worse prognosis of PCa [192]. IGF2BP2 promotes the upregulation of PCAT6 in an m^6^A-dependent manner. In addition, PCAT6 enhances IGF1R mRNA stability via the PCAT6/IGF2BP2/IGF1R RNA–protein trimer, thereby upregulating IGF1R expression and promoting PCa bone metastasis and tumor growth [174]. A clinical case study depicted that IGF2BP3 was associated with infiltrative tumor recurrence [193]. IGF2BP3 also binds to cyclic RNA hsa_circ_0003258 in the cytoplasm, enhancing the stability of HDAC4 mRNA, activating the ERK pathway, and triggering EMT to accelerate the transfer of PCa [194].

### Role of m^6^A modification in BCa

#### The role of m^6^A writers in BCa

Several studies have revealed that METTL3, a core component of m^6^A methyltransferase, is significantly upregulated in BCa and contributes to cancer progression. For instance, Han et al. found that METTL3 may have oncogenic effects in BCa by interacting with DGCR8 and positively regulating the pri-miR222/6 process in an m^6^A-dependent manner [195]. MEETL3 downregulation significantly reduced BCa proliferation, invasion, and tumorigenicity in vivo. In contrast, overexpression of METTL3 promoted BCa cell growth, mechanistically triggered by METTL3-mediated m^6^A modification, mediating activation of the AFF4/NF-κb/Myc signaling pathway [196]. Activation of JNK signaling is also associated with increased METTL3 expression in Bca, where knocking down of JNK1 or administration of JNK inhibitors resulted in impairment of c-JUN binding to the METTL3 promoter, thereby reducing the expression of METTL3 and global RNA m^6^A levels, in addition to JNK signaling to suppress PD-L1 mRNA expression abundance, which revealed that METTL3 could promote BCa immune escape [197]. Xie et al. employed MeRIP and found that the METTL3/YTHDF2 m^6^A axis directly degrades the mRNAs of tumor suppressors SETD7 and KLF4 and promotes BCa development, whereas METTL3 depletion contributes to the impairment of cancer proliferation and metastasis [198]. The METTL3 is also associated with BCa cell adhesion; reported by Jin et al. that upregulation of the adhesion factor ITGA6 correlated with increased METTL3 expression in human BC tissues, and higher ITGA6 expression in patients was associated with lower survival rates. Mechanistically, m^6^A is highly enriched in ITGA6 transcripts, and increased m^6^A methylation of the ITGA6 mRNA 3 promoter promotes translation of ITGA6 mRNA by binding to m^6^A readers YTHDF1 and YTHDF3 [199]. Similarly, Wang et al. found that removing METTL3 in the bladder uroepithelium attenuated bladder carcinogenesis and tumor angiogenesis. Furthermore, conditional knockdown of METTL3 in BCa stem cell populations inhibits Bca progression. Combining transcriptome sequencing and methylation RNA immunoprecipitation sequencing results, we found that METTL3 deletion reduced the abundance of tyrosine kinase endothelial (TEK) and vascular endothelial growth factor A (VEGF-A) m^6^A peaks in specific regions. In addition, the deletion of METTL3 reduced the mRNA and protein levels of both TEK and VEGF-A in vitro. Taken together, METTL3-mediated m^6^A modification is required for the activation of TEK-VEGF-A-mediated tumor progression and angiogenesis [200]. Yang et al. reported that METTL3 and CDCP1 expression was upregulated in BCa tissues, and their expression levels were correlated with BCa progression, in addition to the METTL3-m^6^A-CDCP1 axis inhibiting chemotransformation and BCa cell growth and progression. This axis synergizes with chemical carcinogens to promote malignant transformation of uroepithelial cells and BCa development [201]. Ying et al. demonstrated that the RCas9-METTL3 system mediates efficient site-specific m^6^A installation on CDCP1 mRNA, thereby promoting BC progression [202]. Long-term exposure to fine particulate matter (PM2.5) is also associated with various cancers, including Bca, which was reported in a study by Liu et al. stating that PM2.5 exposure was significantly associated with increased levels of m^6^A modification in Bca patients and bladder cells, with abnormally upregulated METTL3 expression. METTL3 is also involved in PM2.5-induced m^6^A methylation; enhancing METTL3 expression induces METTL3 promoter hypermethylation and increases the binding affinity of the transcription factor HIF1A mechanistically. Similarly, the BIRC5 was identified as the target gene of METTL3 by m^6^A sequencing (m^6^A- seq) and KEGG analysis. The methylated BIRC5 transcripts are subsequently recognized by IGF2BP3, enhancing its mRNA stability. In particular, PM2.5 exposure promoted m^6^A modification of BIRC5 and its recognition by IGF2BP3. In addition, BIRC5 is involved in BCa proliferation and metastasis, as well as in VEGFA-regulated angiogenesis. It was also revealed that PM2.5 exposure exerts epigenetic regulation on BCa through the HIF1A/METTL3/IGF2BP3/BIRC5/VEGFA axis [203].

In other "writer" studies, Gu et al. found lower expression of METTL4 in BCa, where knocking down METTL4 promoted BCa proliferation, self-renewal, metastasis, and tumor initiation. In contrast, the opposite effects were observed in the case of overexpressed METTL4 [204]. In addition, Teixeira et al. found that METTL14 knockdown disrupted the remaining methyltransferase complex, decreased m^6^A abundance, and reduced tumor aggressiveness, including decreased cell invasion and migration capacity and increased apoptosis. Furthermore, in vivo*,* METTL14 knockdown also reduces tumor size [205]. The biological role played by METTL14 in BCa needs to be supported by more studies.

#### Role of m^6^A erasers in BCa

Recent studies have identified FTO as a critical factor for the oncogenic effects of BCa. It has been shown that FTO exhibits oncogenic effects in BCa by regulating the expression of cell cycle protein-dependent kinase (CDK6), which is closely related to the cell cycle, and mechanistically promotes cancer cell proliferation and invasion in BCa through the FTO/miR-576/CDK6 pathway [206]. Song et al. found that post-translational deubiquitination of USP18 upregulates FTO protein expression, whereas FTO promotes the occurrence and development of BCa through its demethylase activity on PYCR1 and stabilizes its transcript [207]. In addition, FTO regulates the MALAT/miR-384/MAL2 axis through m^6^A RNA modification, leading to Bca [208]. Jin et al. displayed that METTL3 and ALKBH5 altered cell adhesion by regulating ITGA6 expression in BC cells, suggesting an oncogenic effect of m^6^A-modified ITGA6 and its regulatory mechanism on BCa initiation and progression. Simultaneously, the downregulation of ALKBH5 promotes BCa cell proliferation, invasion, and migration [209].

#### Role of m^6^A readers in BCa

All three YTHDF proteins in BCa were oncogenic. For instance, YTHDF1/YTHDF3 promotes tumor growth and progression by recognizing m^6^A-modified ITGA6 mRNA and promoting its translation [199]. In addition, YTHDF1 promotes BCa cell proliferation through the METTL3/YTHDF1-RPN2-PI3K/AKT/mTOR regulatory axis [210]. YTHDF2 promotes tumorigenesis by accelerating the degradation of oncogenes SETD7 mRNA and KLF4 mRNA in BCa [198], in addition to mediating the downregulation of oncogene RIG-I levels through m^6^A modification, thereby promoting BCa cell proliferation. Xie et al. found that IGF2BP1 binds to circPTPRA in the cytoplasm of BC cells and that ectopic expression of circPTPRA abolishes the promotion of BCa cell growth and metastasis induced by IGF2BP1 [211]. Huang et al. also reported elevated expression of IGF2BP3 in BCa tissues, where its overexpression significantly promoted the cell cycle and BC cell proliferation by activating the JAK/STAT signaling pathway and inhibiting apoptosis [212].

### Role of m^6^A modification in RCC

#### The role of m^6^A writers in RCC

There is now substantial evidence that m^6^A modifications mediated by different regulatory factors affect RCC progression by inhibiting or promoting its effects. Zhu et al. found that METTL3 expression was significantly higher in RCC tissues than in adjacent normal tissues and that cell viability, migratory capacity, invasive capacity, and in vivo tumor formation were significantly inhibited when METTL3 was depleted [213]. It was observed that METTL3 promotes RCC tumor progression, migration, and tumor spheroid (stem cell-like tumorigenic cell) formation through m^6^A modification-mediated ABCD1 translation [214]. It has been reported that decreasing MEEL14 reduces m^6^A modification of BPTF, which enhances mRNA stability and protein expression, leading to glycolytic reprogramming and metabolic remodeling in RCC cells [215]. Similarly, reduced METTL14 was revealed to promote the translation of P2X6 mRNA, activate the ATP-P2X6-Ca2 + -p-ERK1/ERK2-MMP9 signaling pathway, translate into cell migration and invasion, and is detrimental to the prognosis of RCC [216]. It was also demonstrated that enhancement of mucin 15 (MUC15) and m^6^A modification of histidine-rich glycoprotein (HRG) mRNA using dCas13b-M3M14 fusion protein inhibited the metastasis of kidney cancer, further confirming the role of METTL3 and METTL14 in kidney cancer and providing a potential intervention strategy [217]. The mir-501-3p-CDK2 axis increases WTAP expression in RCC tissues, enhancing the stability and expression of S1PR3 mRNA in an IGF2BPs-dependent manner, thereby driving renal cell carcinogenesis, metastasis and overall poor survival through the regulation of the PI3K/Akt signaling pathway [218–220]. Zeng et al. found that RBM15 expression was upregulated in RCC cells and tissues, where EP300/CBP-induced acetylation modification of the RBM15 promoter led to enhanced RBM15 expression and enhanced the expression and stability of CXCL11, thereby promoting macrophage infiltration and M2 polarization, leading to cancer progression in an m^6^A-dependent manner [221].

#### The role of m^6^A erasers in RCC

Demethylases are dysregulated in the development and progression of RCC, and these differences are often associated with metastasis, survival, and poor prognosis. The transcript and protein levels of demethylases, including FTO and ALKBH5, differ across subtypes of renal cancer. A previous study demonstrated that upregulated expression of ALKBH5 regulates AURKB mRNA expression in an m^6^A-dependent manner, thereby promoting cancer cell proliferation, migration, and invasion, leading to increased RCC volume and poor prognosis [222]. However, dcas13b-ALKBH5-induced demethylation of procollagen lysine and 2-ketoglutarate-5-dioxygenase 2 (PLOD2) mRNA has been shown to play an inverse role [223]. Similarly, FTO has been shown to have opposite roles in RCC. It was found that m^6^A modification levels are reduced in RCC and are closely associated with autophagy, and silencing FTO impairs RCC growth and metastasis. Mechanistically, SIK2 was identified as a functional target of m^6^A-mediated autophagy, thus prompting a conserved and important role of FTO in inhibiting autophagy and promoting tumorigenesis through an m^6^A-IGF2BP2-dependent mechanism [224]. However, the inconsistent roles of FTO and ALKBH5 in RCC are most likely due to the neglect of tumor heterogeneity, different RCC subtypes, or enzyme interaction [225–228].

#### The role of m^6^A readers in RCC

A previous study reported that m^6^A methylation recognition enzymes are commonly dysregulated in RCC [229]. For example, Wu et al. identified hnRNPC as a potential target biomarker for RCC by examining the in vitro expression level, survival outcome, PPI network, functional enrichment, immune cell infiltration, and single-cell analysis. hnRNP promotes RCC cell proliferation and migration [230]. As known m^6^A readers, IGF2BPs proteins are upregulated in most cancers and mediate enhanced m^6^A-modified mRNA stability. Ying et al. found that early EGR2 transcription factors increase IGF2BPs expression in kidney cancer. Similarly, igF2BPs enhance the stability of sphingosine 1-phosphate receptor 3 (S1PR3) mRNA by enhancing the m^6^A-dependent expression of IGF2BPs, which regulates S1PR3 expression in an m^6^A-dependent manner and promotes renal tumorigenesis through the PI3K/AKT pathway [220]. IGFBP1 promotes RCC tumor energy metabolism, including glucose uptake, lactate survival, and extracellular acidification rate, by recognizing the m^6^A modification site on LDHA mRNA and enhancing its mRNA stability, thereby accelerating tumor energy metabolism [231]. IGF2BP3 binds to DDRMR and specifically enhances IGF2BP3 activity against target genes, including the cell cycle kinase CDK4 and three extracellular matrix components (COL6A1, LAMA5, and FN1), in an m^6^A-dependent manner, thereby stabilizing these genes. Similarly, DDRMR and IGF2BP3 promote the transition of ccRCC cells from G1 to S phase, thereby promoting cell proliferation [232]. In the YTH family, YTHDF2 is associated with immune infiltration and RCC [233, 234].

### Role of m^6^A modification in TC

METTL3 is an important methylation enzyme that is associated with cancer progression. Luo et al. found that METTL3 is involved in the proliferation, migration, and invasion of TC cells by regulating the expression of EMT-related genes and may also play a role in activating tumor immune responses in TC [235]. METTL3 also regulates the mRNA stability of transcription factor activation enhancer binding protein 2C (TFAP2C) through m^6^A modification, leading to cisplatin resistance in TC [236]. Knockdown of VIRMA, another methyltransferase, results in disruption of the remaining methyltransferase complex and decreased m^6^A abundance, resulting in reduced overall invasiveness of TC (reduced cell viability, tumor cell proliferation, migration, and invasion) and increased sensitivity to cisplatin treatment, demonstrated both in vitro and in vivo [237]. The functions of the three different methylesterases of m^6^A in TC are still scarcely studied, requiring further confirmatory studies.

## Clinical potential of m^6^A in urological cancers

The dynamic and reversible roles of RNA modifications in pathology have been recognized. The development of different tumor types is associated with different functions of RNA modifications. With the development of transcriptomics, failure of early diagnosis and poor prognosis due to ineffective treatment remain prevalent in the management of patients with cancer, including urological tumors. Therefore, these regulatory proteins have great potential for early diagnosis, improved prognosis, and treatment of urological cancers as new diagnostic, prognostic, and therapeutic targets.

### m^6^A-modified regulators as new biomarkers for urological cancers

Various studies have shown a correlation between m^6^A methylation and cancer initiation and progression. Many recent studies have identified m^6^A methylation as a diagnostic and prognostic-related biomarker for cancer. For example, Ji et al. found that IGF2BP3, hnRNPA2B1, and METTL14 are significantly associated with PCa prognosis [238]. RCC patients with higher IGFBP3 expression also depict longer metastasis-free and overall survival [239]. Cheng et al. found that WTAP expression was significantly higher in the BC group than in the control group by comparing fresh BCa and normal bladder mucosa specimens, and a significant difference in the risk of disease recurrence was observed between patients with negative and positive WTAP protein expression levels [240]. In addition, METTL3, ALKBH5, IGF2BP3, and FTO levels are closely associated with the prognosis of BC patients [195, 208, 209, 212]. In RCC, the expression levels of ALKBH5 and FTO are associated with shorter overall and cancer-specific survival after nephrectomy and can be used as prognostic biomarkers [226]. Other methylation regulators, including METTL3, WTAP, and IGFBP1/2/3, are also associated with RCC prognosis [241]. Cong et al. screened a database for YTHDF1, RBM15, IGFBP1, ZC3H13, and regulatory factors that play an important role in the prognosis of TC patients [242].

### Potential drug and therapeutic strategies based on m^6^A-modified regulators

Given that RNA modifications, especially m^6^A modifications, play an important biological role in various types of cancers, developing targeted drugs based on m^6^A modifications has become a promising strategy. For example, an inhibitor based on METTL3, STM2457, developed as a treatment option for hematological malignancies, blocks the proliferation and colony formation of MOLM-13 cell lines and promotes apoptosis without affecting normal hematopoietic function. In in vivo studies, STM2457 inhibited the proliferation of acute myeloid leukemia (AML) in patient-derived xenograft models and leukemia mouse models [243]. Cheng et al. developed two potent FTO inhibitors, FB23 and FB23-2, which directly bind to FTO and selectively block its m^6^A demethylase activity [244]. Subsequently, they developed two other FTO inhibitors, CS1 and CS2, which exhibited strong antitumor effects in various cancers. In leukemia, FTO inhibitors block the FTO/m^6^A/MYC/CEBPA signaling axis, inhibiting the autologous renewal of tumor stem cells and the expression of the immune checkpoint LILRB4 and immune evasion, thereby enhancing the cytotoxicity of T cells [245]. Many studies have demonstrated that m^6^A regulators are important in regulating the tumor immune microenvironment. For example, YTHDF2 acts as an m^6^A reader that isolates m^6^A-circRNA and plays a vital role in suppressing intrinsic immunity [246]. Han et al. demonstrated that YTHDF1 exerts antitumor effects through m^6^A methylation in dendritic cells (DCs). Antigen-specific CD1^+^ T-cell antitumor responses are significantly enhanced in YTHDF1-deficient mice, significantly improving the therapeutic effect of PD-L1 checkpoint blockade [247]. Cheng et al. reported that hnRNPC suppresses PCa tumor immunity by increasing Treg cell activation and suppressing effector CD8 T-cells. Epigenetic alterations can also lead to resistance to chemotherapy and radiotherapy, limiting their efficacy [248], and changes in the expression level of METTL3 made PCa cells resistant to AR antagonists [249]. A newly identified circRNA circ0008399 binds to WTAP and reduces cisplatin sensitivity in BCa by regulating target RNA expression through m^6^A modification, suggesting the potential therapeutic value of targeting this axis [250]. Chen et al. found that n6-methyladenosine-modified TRAF1 promotes resistance to sunitinib in renal cancer by regulating apoptosis and angiogenesis in a METTL14-dependent manner [251]. I*n vivo* and in vitro assays further demonstrated that the knockdown of VIRMA resulted in an enhanced responsiveness of TC to cisplatin and a significant increase in DNA damage [237].

## Problems in the clinical application of RNA modification

With the recent development of effective inhibitors against m^6^A-modified proteins, progress in this area has been demonstrated. However, their use as therapeutic agents remains challenging, mainly due to the lack of consistent and consolidated proof. For example, the oncogenic or pro-carcinogenic nature of the aberrant deposition of m^6^A in different types of cancer. Although several methylases and Ψ-synthases have been identified that have been found to be aberrantly expressed in cancer, it remains unclear whether they can be effective targets for cancer therapy. In addition, little is known about how the binding of their writers, erasers, and readers affects RNA metabolism and the fate of tumor cells. However, it is undeniable that the availability and selective inhibitors of the 3D structures of most of these regulatory factors have been discovered, suggesting that inhibition of these enzymes is achievable [252]. For example, for the m^5^C methylases azacytidine and decitabine are cytidine analogs that inhibit any m^5^C methylase and have been approved for clinical use in hematologic malignancies [253]. However, these inhibitors lack specificity and should be used with caution.

From a clinical perspective, Ψ may have potential as an early biomarker. Large amounts of Ψ have been detected in urine of patients with colon cancer, prostate cancer ovarian cancer, and salivary metabolites of patients with oral squamous cell carcinoma, suggesting that it could be used as a potential biomarker for early cancer diagnosis in non-invasive biopsies [254–257]. Overall, there are still many problems with the clinical application of RNA modification, but this does not prevent us from affirming the development of small-molecule inhibitors targeting RNA modification sites and RNA modifying enzymes, which will provide a more targeted approach to cancer therapy. We need more relevant studies to validate and further explain the specific mechanisms of RNA methylation in cancer and to resolve some conflicting studies.

## Discussion

Currently, the biological functions of m^6^A in regulating urologic cancer mainly focus on proliferation, invasion, migration, angiogenesis, immune escape, and drug resistance [258, 259]. However, tumor metabolic reprogramming, an important cancer feature, has been neglected because of the flexible changes in cancer cell metabolism; it can not only meet the needs of cell growth but also maintain the homeostasis of the tissue environment. Metabolic adaptations are acquired through endogenous and exogenous signaling pathways [260]. Glucose and lipid metabolism are important components of tumor metabolism. Glucose metabolism can be divided into catabolism and anabolism processes. It is well known that energy metabolism is a hallmark of the high invasiveness of cancer cells, including increased glycolytic activity, lactic acid fermentation, and the Warburg effect [259, 261]. The Warburg effect is an important feature of abnormal glucose metabolism in tumors. It can increase glycolysis and glucose uptake and consumption, which causes tumor cells to proliferate differently from normal cells [262].

In urological cancers, specific alterations in many metabolic pathways are also important features [263–265]. For example, RCC is essentially a metabolic disease characterized by reprogramming energy metabolism [266]. In particular, metabolic flow through glycolysis is fragmented, and mitochondrial bioenergetics, oxidative enzymes, and lipid metabolism [266–272]. In this context, the role of m^6^A RNA methylation as a regulator of cancer cell metabolism in urologic tumors is also a topic of discussion. In prostate cancer, glycolysis is not a hallmark of primary prostate cancer and plays a key role only in advanced cancers [175–177]. It has been shown that METTL3 enhances SNHG7 stability by regulating m^6^A modification of SNHG7 and recruits SRSF1 to regulate c-Myc expression, further promoting glycolysis in PCa cells [178]. Amino acids are produced by proteolysis. The metabolism of amino acids in the body occurs mainly through synthesizing nitrogenous substances, such as proteins and peptides, and the decomposition of amino acids to produce α-ketoacids and CO_2_ through deamination and transamination [273]. VHL proteins are recognition sites for HIF family substrates, and targeting the HIF family degrades ubiquitin-mediated proteasomes. In RCC, tumor suppressor VHL deletion is an important marker. Inactivation of VHL leads to activation of VEGF and PDGF and targets the downstream glutamine transporter SLC1A5, promoting metabolic reprogramming of VHL-deficient RCC and selectively reducing the growth of VHL-deficient RCC [227]. Mitochondria are the energy factories of the cell, producing the energy currency ATP for the cell by burning glucose, amino acids, and lipids to perform various life activities. There are also many correlations between mitochondrial metabolism and tumorigenesis [274]. Methylenetetrahydrofolate dehydrogenase 2 (MTHFD2) is a mitochondrial enzyme involved in single-carbon metabolism that regulates the HIF-2α transcriptome, thus influencing RCC progression. Although MTHDF2 is still not clearly defined as a methyltransferase of m^6^A, MTHFD2 expression is significantly elevated in renal cancer cells and regulates the level of m^6^A methylation. mTHDF2 regulates HIF-2α m^6^A methylation and promotes HIF-2α expression, thereby facilitating the metabolic reprogramming of tumor cells [275].

Tumorigenesis and development cannot be supported by the tumor microenvironment, which is mainly characterized by hypoxia, metabolic dysregulation, inflammation, and immune infiltration [276, 277]. Evidence shows that m^6^A mediates biological processes in cancer and stromal cells through splicing, translation, degradation, and export of regulatory factor expression, thus characterizing the TME. m^6^A also plays an important role in the complex regulatory network of m^6^A modifications, which in turn affects tumor initiation, progression, and therapeutic responsiveness [278, 279]. For example, RCC is one of the most immune-invasive tumors, and its immune regulation has a profound impact on the prognosis of RCC [280–282]. Whether m^6^A RNA methylation regulates immune infiltration in TME in urologic cancers and its function is also a question worthy of further exploration.

The treatment of urologic cancers still has many pain points and difficulties. For example, the androgen receptor (AR) plays a crucial role in the pathogenesis of prostate cancer and is late in treatment due to the adaptation of PCa cells to low levels of androgens. The AR system remains continuously activated, leading to the inevitable development of destructively resistant prostate cancer. It has been displayed that METTL 3 plays a functional role in this. Roy et al. found that METTL3 expression was higher in AR-expressing PCa cell lines than in AR-negative PCa cell lines, and similar results were obtained at the protein level. This finding suggests a potential interaction between METTL3 and the androgen signaling pathway. Notably, knockdown of METTL3 resulted in increased expression of the AR target gene NKX3.1 and decreased prostate-specific antigen (PSA) expression, suggesting a direct role of METTL3 in AR expression [283]. Knockdown of METTL3 also resulted in the elevation of key regulators, such as KDm^1^A, which is involved in PCa initiation and progression and regulates AR expression and function [284–286]. Therefore, further studies on the effects of METTL3 on the entire androgen signaling pathway are needed. Because of the role that m^6^A methylation plays an important role in the splicing process [287, 288], future studies could also explore whether METTL3 plays a role in the progression of PCa to CRPC due to the AR splicing process. m^6^A is expected to serve as a biomarker for prostate cancer diagnosis and clinical intervention.

Overall, RNA modifications are involved in the biological functions of many types of cancer. The multiple functions and mechanisms of m^6^A modification involved in developing urological cancers require further investigation.

## Conclusion and prospects

RNA modifications play an important role as key post-transcriptional regulators of gene expression, and the functional network of interactions involves multiple domains such as metabolism, epigenetics, chromatin remodeling, and the immune system. Great breakthroughs have been made in the transcriptome study, including more than 170 chemical modifications of coding and non-coding RNAs; however, most have focused on the biological functions of one or a few RNA modifications. This article describes the common RNA modifications under study; however, other types of RNA modifications remain to be explored.

Compared to other RNA modifications, m^6^A modifications are the most abundant and well-characterized internal modifications in mRNA, which prompted the desire to fully understand their specific modes of action. Unlike DNA methylation and histone modifications, m^6^A RNA modifications target almost all transcripts and extensively regulate their processing, stability, and translation levels [35, 119]. Overall, basic and clinical studies have demonstrated that m^6^A modifications can dynamically and reversibly regulate biological changes in cancer cells. This has provided new therapeutic ideas and directions for treating urological cancer. This paper reviews the role of RNA modifications, especially m^6^A modifications, on the proliferation, migration, and invasion of urological cancers and summarizes some potential clinical applications of current m^6^A modifications. Although the study of m^6^A methylation modification in urological cancers is still at an early stage, it will be significant and valuable to elucidate the mechanism of m^6^A methylation modification in the development of urological cancers, screen and explore potential targets, and conduct preclinical trials, which will help establish new therapeutic strategies.

As research continues, m^6^A modifications are becoming more evident. However, it still faces certain limitations, as most studies currently focus on the role played by only a few regulatory factors, ignoring the importance of the overall study and their synergistic role, which requires further research. Second, many studies target the regulation of downstream factors by m^6^A methylation and exert oncogenic effects, while studies exploring the upstream factors that can lead to abnormal m^6^A methylation are rare. Third, in the study of m^6^A modification in urologic cancers, most researchers have focused on the biological functions of proliferation, migration, and invasion. At the same time, little or no exploration has been done on tumor angiogenesis, glycolipid metabolism, and the maintenance of tumor stem cells, which requires further study. Finally, there is a lack of large-scale multicenter clinical trials to verify its feasibility.

## Data Availability

Not applicable.
